# Treatment of cheese whey wastewater by electrochemical oxidation using BDD, Ti/RuO_2_-TiO_2_, and Ti/RuO_2_-IrO_2_-Pt anodes: ecotoxicological and energetic evaluation

**DOI:** 10.1007/s11356-025-36174-0

**Published:** 2025-03-04

**Authors:** Imen Souli, Annabel Fernandes, Ana Lopes, Inês Gomes, Alexandra Afonso, Lazhar Labiadh, Salah Ammar

**Affiliations:** 1 Laboratoire de Recherche Matériaux, Electrochimie Et Environnement LRM2E (LR24ES18), Faculté Des Sciences de Gabès, Université de Gabès, Cité Erriadh, 6072 Gabès, Tunisia; 2 Fiber Materials and Environmental Technologies (FibEnTech-UBI), Universidade da Beira Interior, R. Marquês de D’Ávila E Bolama, 6201-001 Covilhã, Portugal; 3 Department of Applied Sciences and Technologies, Instituto Politécnico de Beja, Escola Superior Agrária de Beja, 7800-309 Beja, Portugal; 4 Department of Chemistry, Universidade da Beira Interior, Rua Marquês D’Ávila E Bolama, 6201-001 Covilhã, Portugal

**Keywords:** Cheese whey wastewater, Anodic oxidation, Boron-doped diamond, Mixed metal oxides, Biodegradability, Ecotoxicity

## Abstract

The effectiveness of boron-doped diamond (BDD) and titanium metal-mixed oxides (Ti/MMO: Ti/RuO_2_-TiO_2_ and Ti/RuO_2_-IrO_2_-Pt) anodes to treat cheese whey wastewater (CWW) by electrochemical oxidation (EO) was evaluated. The results show that EO with BDD is effective in the removal of organic compounds. Conversely, Ti/MMO anodes exhibit higher removals of nitrogenated compounds. After 8 h of EO treatment at an applied current density of 500 A m^−2^, the biodegradability index increased from 0.55 to 0.81 with the BDD anode, while with Ti/MMO only reached 0.64. The acute toxicity of the CWW, before and after treatment, was assessed with the model organism *Daphnia magna*. The use of BDD showed favorable outcomes, leading to a reduction in ecotoxicity, which changed the CWW classification from “very toxic” to “toxic,” very close to the “non-toxic” level. Contrarywise, the use of Ti/MMO anodes led to an escalation of potentially harmful substances in the treated effluent. Still, Ti/MMO anodes provide the most favorable energy consumption when operating at current densities equal to or below 100 A m^−2^. While both Ti/RuO_2_-TiO_2_ and Ti/RuO_2_-IrO_2_-Pt exhibit similar performance, the effectiveness of Ti/RuO_2_-TiO_2_ is somewhat lower.

## Introduction

The dairy industry is a sector within the agrofood industry that has developed in numerous nations because of the increased demand for milk and its derived products, including cheese, yogurt, and butter (Ahmad et al. 2019). The European Union led global cheese production in 2023, accounting for 37% of the total, as reported by the USA Department of Agriculture (USA Department of Agriculture 2023). Following closely is USA, contributing 29% to the worldwide production. This industry is water-intensive and generally requires large quantities of high-quality water. For instance, cheese production in European dairies may require up to 3.7 L of water per L of milk processed, mainly due to cleaning and rinsing needs (Chamberland et al. 2020).

The raw wastewaters discharged from this industry contain heavy loads of organic matter, salinity, nutrients, solids, oils, and fats (Guerreiro et al. 2020). Therefore, direct disposal of these effluents without any treatment may have significant and limitless negative consequences for the ecosystem and public health (Ahmad et al. 2019; Valta et al. 2017). The main contributors of organic load to the dairy industry waste effluents are carbohydrates, proteins, and fats originating from the milk (Yavuz et al. 2011). The cheese whey wastewater (CWW) ecotoxicity can be attributed to the carbohydrates, proteins, and lipids content and cleaning operations (Danalewich et al. 1998; Cruz-Salomón et al. 2020). Moreover, cheese whey is regarded as the most important pollutant in dairy wastewater because of the high organic load and the significant volume generated (Carvalho et al. 2013).

CWW requires treatment methods adaptable to its complex nature and allowing the complete elimination of pollutants. Biological methods are commonly employed to treat dairy wastewater (Demirel et al. 2005; Carvalho et al. 2013). Nevertheless, aerobic biological processes are extremely energy-intensive (Kushwahaet al. 2010), whereas effluents treated by anaerobic biological processes require additional treatment because they partly convert the nutrient (Montuelle et al. 1992). Moreover, chemical compositions and the high toxicity of CWW may complicate the biological treatment (Danalewich et al. 1998; Ganzenko et al. 2014; Liu et al. 2010). CWW treatment also entails the utilization of physicochemical methods. These techniques involve membrane technologies, coagulation-flocculation, and precipitation (Reig et al. 2021; Rivas et al. 2010; Prazeres et al. 2020). However, these traditional processes are accompanied by several disadvantages, such as the use of expensive reagents and the production of sludge (Das and Chen 2024).

Several electrochemical processes have been applied to CWW treatment (Abdelhay et al. 2019; Borbón et al. 2014; Elia et al. 2023; Katsoni et al. 2014; Markou et al. 2017; Tirado et al. 2018). Electrochemical oxidation (EO) is widely used for wastewater treatment and environmental pollution remediation (Ganiyu et al. 2021). This technology has gained great attention because of its versatility, ease of operation, and, especially, its high efficiency in the degradation of recalcitrant compounds, particularly when boron-doped diamond (BDD) anodes are used (Fu et al. 2023; Souli et al. 2023). BDD is considered the most efficient for the EO process due to its high potential to generate large quantities of hydroxyl radicals, which are non-selective oxidants with high oxidation potential, as well as different secondary oxidants (Fu et al. 2023). Alongside BDD, titanium-based mixed metal oxides (MMO) are auspicious anode materials due to the titanium substrate’s excellent stability and high catalytic activity, conferred by the modification of the anode surface through a combination of several metal oxides (Qiao and Xiong 2021). The performance of the Ti/MMO electrodes is highly dependent on the material of the electrocatalytic layer coating the Ti substrate (Wang et al. 2020). Depending on that, the hydroxyl radicals formed from water electrolysis can be either chemisorbed (Ti/MMO_x+1_), exhibiting reduced reactivity due to its strong and stable bond with the anode surface, or physisorbed (Ti/MMO(•OH)), leading to weakly bounded •OH radicals that, being highly reactive, promote the complete mineralization of the organic compounds (Zarei et al. 2023). In addition to the hydroxyl radicals, other oxidizing species can be formed during the EO process, such as active chlorine species when the wastewater is rich in chloride. According to the literature, RuO_2_ is a good electrocatalyst for chlorine evolution (Wang et al. 2020). Conversely, an IrO_2_ interlayer enhances the generation of physisorbed hydroxyl radicals (Aguilar et al. 2018). Ti-based RuO_2_ and IrO_2_ have been widely studied due to their low potential for oxygen evolution reaction and promising results in wastewater remediation (Zarei et al. 2023).

The literature studies reporting the EO treatment of dairy wastewater used mostly BDD anodes (Abdelhay et al. 2019; Elia et al. 2023; Katsoni et al. 2014; Tirado et al. 2018). Nonetheless, alternative anode materials have demonstrated promising results (Borbón et al. 2014; Markou et al. 2017). In a study performed by Markou et al. (2017), who investigated the EO treatment of aerobically pretreated dairy wastewater, an almost complete organic load removal was found using an IrO_2_/Ti anode. Borbón et al. (2014) investigated the utilization of a Ti/IrO_2_-Ta_2_O_5_ anode for the treatment of dairy wastewater, comprising an electrocoagulation step followed by EO, achieving 98% removal of total organic carbon. It should be noted that the EO treatment of raw dairy wastewater is barely described in the literature, being that, in most of the studies, EO was applied as a post-treatment, following biological or electrocoagulation steps, which introduce sludge formation in the treatment, highly undesirable when considering wastewaters management (Borbón et al. 2014; Elia et al. 2023; Katsoni et al. 2014; Markou et al. 2017; Tirado et al. 2018). Furthermore, the change in the toxicity of treated wastewater is one of the important considerations for the success of the application of EO treatment (Wilk et al. 2022). It is important to note that only a limited number of studies have focused on the evaluation of toxicity of treated CWW by electrochemical oxidation. Katsoni et al. (2014) applied EO, using a BDD anode, to treat an anaerobically treated cheese whey, and they found that the ecotoxicity of treated wastewater towards *Artemia salina* increased.

The present study is aimed at evaluating the performance of different anode materials, BDD, Ti/RuO_2_-TiO_2_, and Ti/RuO_2_-IrO_2_-Pt, for the EO treatment of raw CWW. The removal of CWW pollutants was assessed at different applied current densities. The biodegradability index was determined before and after EO treatment. An ecotoxicological and energetic evaluation was also performed, providing a comparative study on the final toxicity towards *Daphnia magna* of the CWW treated by different anode materials.

## Materials and methods

### Cheese whey wastewater

The raw CWW was collected in February 2023 from a homogenization tank in a cheese factory in Beja district (Portugal). The cheese whey wastewater comprised cheese whey (from cheese production) and wastewater from washing equipment and tanks. The sample’s pH (11.4 ± 0.3) and temperature (12.4 ± 0.5 °C) were measured at the collection point. Immediately after collection, the CWW sample was characterized and stored at − 20 °C until its use. Before utilization in the EO experiments, the CWW sample was again characterized. Table 1 presents the average CWW characterization obtained from the different determinations performed.

**Table 1:** Characterization of the raw cheese whey wastewater sample utilized in the EO experiments

Parameter	Mean value (± SD)
Chemical oxygen demand (g L^−1^)	3.9 ± 0.1
Biochemical oxygen demand (g L^−1^)	2.15 ± 0.05
BOD_5_/COD	0.55
EC_50_−48 h (%)	4.62
Toxic units	21.7
Total dissolved carbon (g L^−1^)	1.26 ± 0.09
Dissolved organic carbon (g L^−1^)	1.13 ± 0.07
Dissolved inorganic carbon (g L^−1^)	0.13 ± 0.01
Total dissolved nitrogen (mg L^−1^)	91 ± 7
Chloride (mg L^−1^)	800 ± 9
pH	11.8 ± 0.1
Electrical conductivity (mS cm^−1^)	5.9 ± 0.4
Color (visual)	greenish-yellow

### Electrooxidation experiments

The electrochemical assays were run in batch mode with stirring (200 rpm) for 8 h, utilizing an undivided cylindrical glass cell containing 150 mL of CWW sample. A BDD electrode (NeoCoat) and two different MMO electrodes, Ti/RuO_2_-TiO_2_ (Insoluble Anode Technology) and Ti/RuO_2_-IrO_2_-Pt (Qixin Titanium), were used as anode. As cathode, a stainless-steel plate was utilized. The anode and the cathode, with an immersed area of 10 cm^2^ each, were placed in parallel, centered in the electrochemical cell, with an inter-electrode gap of 0.5 cm. The anodic current densities (*j*) studied were 50, 100, 300, and 500 A m^−2^, utilizing a GW, Lab DC, model GPS-3030D (0–30 V, 0–3 A) power supply. All the assays were run in duplicate. Reproducibility was observed for all the experimental conditions evaluated.

#### Analytical methods

Chemical oxygen demand (COD) analysis followed the closed reflux and titrimetric methods adjusted for samples containing high chloride concentration (Fernandes et al. 2019). Biochemical oxygen demand (BOD_5_) was determined using the respirometric method described elsewhere (Fernandes et al. 2019).

The ecotoxicity towards *Daphnia magna* was evaluated following the OECD guideline 202 (2004), utilizing a Daphtoxkit F microbiotest, DM081222. EC_50_, corresponding to the concentration responsible for 50% of immobilization, was obtained from the standard data processing method Daphtoxkit F spreadsheet. The ecotoxicity results expressed as toxicity units, TU, were calculated through Eq. (1) (Pablos et al. 2011).

1 $$\text{TU }=\frac{100}{(\%{\text{EC}}_{50})}$$

Total dissolved carbon (TDC), dissolved organic carbon (DOC), dissolved inorganic carbon (DIC), and total dissolved nitrogen (TDN) were analyzed by a Shimadzu TOC-VCPH analyzer combined with a TNM-1 unit. Previously to the analysis, samples were filtered through 1.2-μm glass microfiber membranes.

According to the procedure described elsewhere, chloride concentration was obtained through ionic chromatography (Fernandes et al. 2019). Electrical conductivity (EC) and pH were measured with a Mettler Toledo conductivity meter SevenEasy S30K and a HANNA pH meter HI 931400, respectively. Analyses were performed in triplicate.

The instantaneous current efficiency (ICE) was calculated using Eq. (2), where COD is in g dm^−3^, *t* is the electrolysis time (s), *F* is the Faraday constant (96,485 C mol^–1^), *V* is the sample volume (dm^3^), *I* is the applied current (A), and 8 is the oxygen equivalent mass. Determination of ∂COD/∂t was performed by adjusting polynomial equations to the COD decay with time and calculating the corresponding derivatives.

2 $$\text{ICE }\left(\%\right)=\frac{\partial \text{COD}}{\partial \text{t}}\frac{100\times F\times V}{8\times I}$$

The electric energy per order (*E*_EO_, in kWh m^−3^ order^−1^), defined as the electric energy required to degrade a contaminant by one order of magnitude in a unit volume of contaminated water (Bolton et al. 2001), was calculated using Eq. (3), where *U* is the cell voltage, in *V*, *I* the current intensity, in *A*, Δ*t* the assay duration, in *h*, *V* the CWW volume, in *L*, and COD_i_ and COD_f_ are the initial (before EO treatment) and final (after EO treatment) COD of the sample, in mg L^−1^.

3 $${E}_{\text{EO}}=\frac{U\times I\times \Delta t}{V\times \text{log}{(\text{COD}}_{i}/{\text{COD}}_{f})}$$

## Results and discussion

The CWW characterization (Table 1) shows that, although the biodegradability index (0.55), given by the BOD_5_/COD ratio, is close to the “fairly biodegradable” classification (> 0.6) (Abdalla and Hammam 2014), the toxicity towards *Daphnia magna* (21.7 TU) classifies the CWW as “very toxic” (Pablos et al. 2011). According to the literature, CWW ecotoxicity can be ascribed to the carbohydrates, proteins, and lipid content (Cruz-Salomón et al. 2020).

**Fig. 1:**
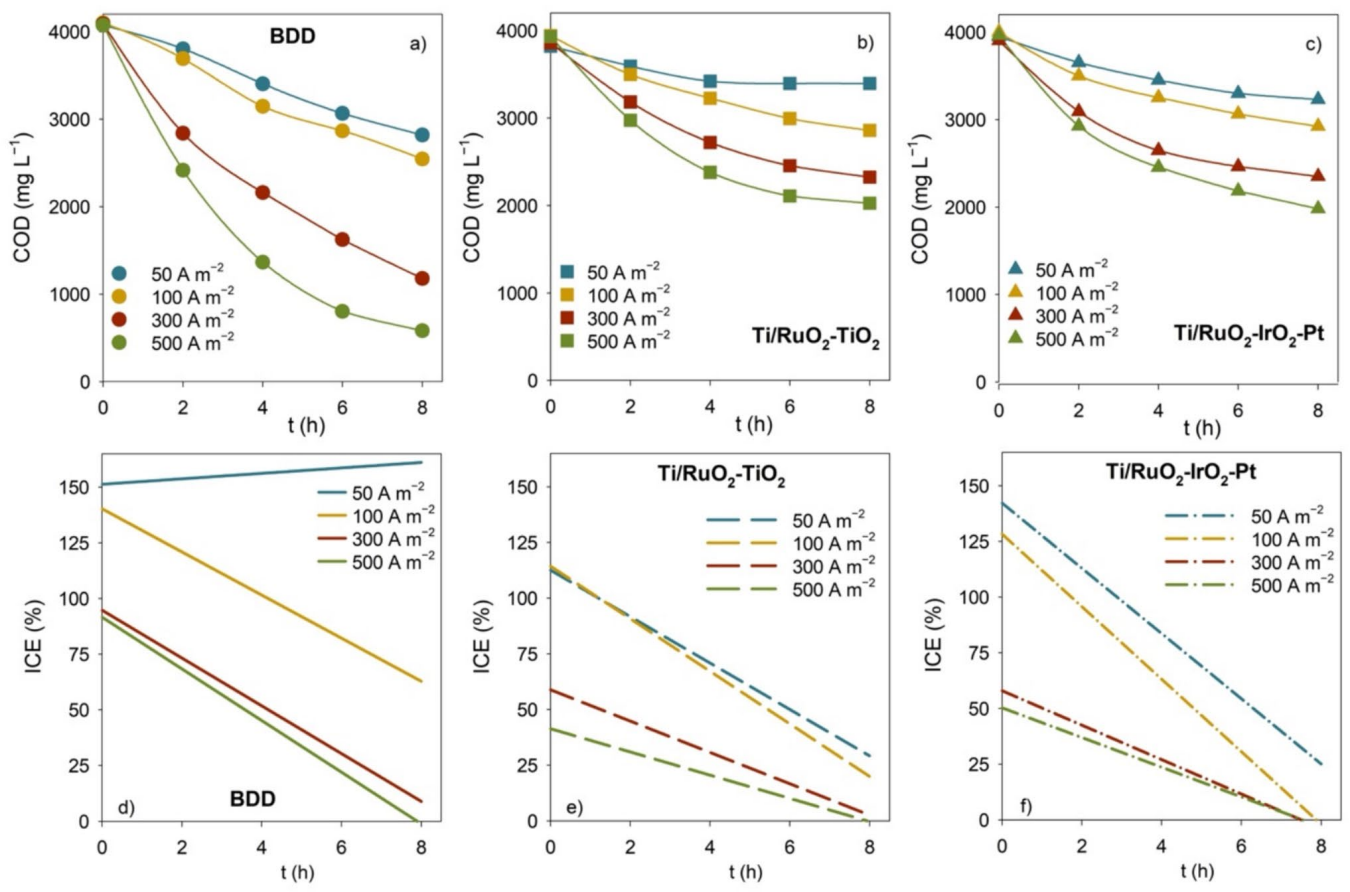
Variation with time of **a–c** COD and **d–f** ICE for the EO experiments performed with BDD, Ti/RuO_2_-TiO_2_, and Ti/RuO_2_-IrO_2_-Pt anodes at different applied current densities

To evaluate the performance of BDD, Ti/RuO_2_-TiO_2_, and Ti/RuO_2_-IrO_2_-Pt anodes in the EO treatment of CWW, experiments utilizing these anodes were run at different current densities. Figure 1 presents the evolution of COD and ICE (calculated using Eq. (2)) for each of the experimental conditions assayed. The BDD anode attained the highest COD removal for all the applied *j*. Ti/RuO_2_-TiO_2_ and Ti/RuO_2_-IrO_2_-Pt presented similar performance, with Ti/RuO_2_-IrO_2_-Pt attaining slightly higher COD removals. An increase in the COD removal rate with *j* was observed for all the anode materials, due to the enhanced formation of •OH and other oxidizing active species that boosted the oxidation of the organic compounds. Contrarywise, ICE results showed a decrease in the current efficiency with the increase in *j*, indicating that, for the highest *j* values, the electrolysis is under mass-transport control, which agrees with the exponential trend of the COD removal at the highest current densities applied (Martínez-Huitle and Andrade 2011). For all the anode materials, at lower current densities (50 and 100 A m^−2^), ICE values above 100% were observed at the beginning of the experiments, which, according to Bagastyo et al. (2021), suggests that the pollutants’ oxidation was governed by an indirect oxidation mechanism.

In general, as the COD decreased during the experiments, the ICE diminished, due to the lower availability of pollutants to be oxidized and the consequent increase of parasitic reactions (e.g., Eqs. (4) and (5)) (Labiadh et al. 2019).

4 $$2\text{ BDD}\left(\cdot \text{OH}\right)\to 2\text{BDD}+{\text{O}}_{2}+2 {\text{H}}^{+}+2 {\text{e}}^{-}$$

5 $${3\text{ H}}_{2}\text{O }\to {\text{O}}_{3} + {6\text{ H}}^{+} + {6\text{ e}}^{-}$$

However, for the experiments run with BDD at the lowest *j* (50 A m^−2^), a slight increase in ICE with time was observed, with ICE values always above 150%. This behavior can be explained by the occurrence of chain reactions involving the formation of radical species, as described by Kapałka et al. (2008a, b). According to these authors, the amount and nature of intermediates formed during EO with BDD anodes strongly depends on *j*: electrolysis under current control results in the formation of a significant number of intermediates; electrolysis under mass-transport control results in CO_2_ as the only final product, with practically no formation of intermediates.

Among the three anodes studied, BDD presented the highest ICE values for all applied *j*, attributed to the higher O_2_ overvoltage and consequent higher oxidation power of this anode material (Kapałka et al. 2008a). Ti/RuO_2_-IrO_2_-Pt presented slightly higher ICE values than Ti/RuO_2_-TiO_2_, especially in the first hours of experiments, indicating that Ti/RuO_2_-TiO_2_ may have a stronger electrode-hydroxyl radical interaction, resulting in a lower chemical reactivity for organic oxidation. This better performance of Ti/RuO_2_-IrO_2_-Pt can be ascribed to its IrO_2_ interlayer, which, according to the literature, enhances the generation of physisorbed hydroxyl radicals (Aguilar et al. 2018), and/or to its Pt layer, which prevents the inactivation of the electrode, increasing its lifetime (Fernandes et al. 2014).

**Fig. 2:**
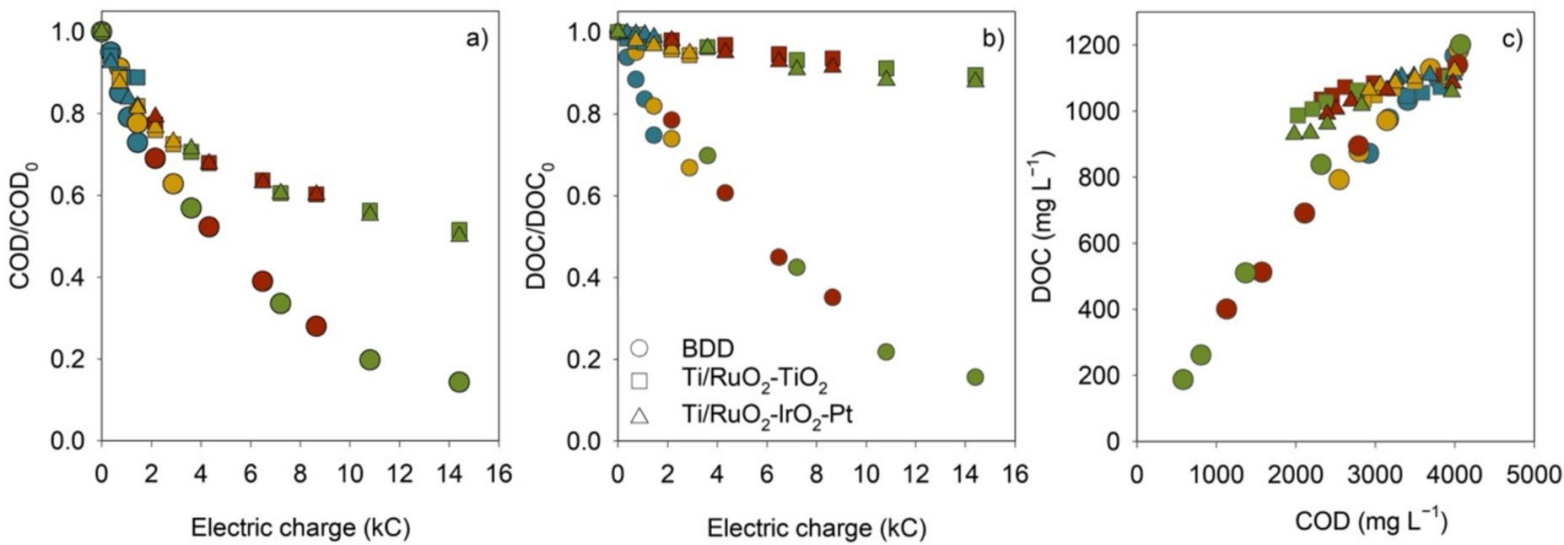
Decay with the electric charge of normalized **a** COD and **b** DOC for the EO experiments performed with BDD, Ti/RuO_2_-TiO_2_, and Ti/RuO_2_-IrO_2_-Pt anodes at different applied current densities. **c** DOC *vs.* COD evolution along the different EO experiments

Normalized COD decays with the applied charge for each anode (Fig. 2a) show that COD decay follows the same trend independently of the applied *j*.

As inferred from the data in Fig. 2a, Ti/RuO_2_-TiO_2_ and Ti/RuO_2_-IrO_2_-Pt have identical performance in COD removal, presenting the same COD decays with applied charge. For low applied electrical charges (< 2 kC), proximity between COD decays at BDD and Ti/MMO anodes is found. Nevertheless, with the increased applied charge, an increased discrepancy between BDD and Ti/MMO anodes can be observed, with the highest COD decays attained by BDD.

Normalized DOC decays with applied charge (Fig. 2b) show similar findings to COD. However, it can be observed that the DOC removal rates are lower than that of COD, especially in the first hours of experiments, making this difference more noticeable for Ti/MMO anodes. A decline in DOC content signifies that EO mineralizes pollutants and converts organic matter into CO_2_. At the same time, a reduction in COD is associated with the change of chemical species during the process (Dbira et al. 2015). This difference between COD and DOC removal rates is explained by the incomplete oxidation of the organic compounds, being more pronounced at Ti/MMO anodes probably due to their higher porosity, which causes stronger adsorption of the •OH formed through the anodic water discharge (Fernandes et al. 2016). Consequently, the formed hydroxyl radicals (Ti/MMO(•OH)) are less prone to react, being favored by the indirect oxidation of the organic compounds through active chlorine species, such as chlorine and hypochlorite, generated from the direct chloride oxidation (Candia-Onfray et al. 2018). Contrarywise to the oxidation through •OH that favors the organic matter mineralization, the indirect oxidation through active chlorine species favors, according to the literature, their partial oxidation, in agreement with the low DOC removal observed when using Ti/MMO anodes (Indhumathi et al. 2001). When using the BDD anode, known for its capability to generate large quantities of weakly adsorbed hydroxyl radicals, the organic compound oxidation through •OH is favored and, consequently, their complete mineralization, although the oxidation by active chlorine species also takes place in parallel (Rodrigues et al. 2022). In addition, in the case of “active” anodes, MO_x_(•OH) can be oxidized to form MO_x+1_ oxide. Then, MO_x+1_ operates as a go-between in the oxidation process, leading to the partial oxidation of the organic compounds (De Coster et al. 2017; Labiadh et al. 2016). In contrast, at “non-active” anodes (like BDD), adsorbed hydroxyl radicals BDD(•OH) react directly with the oxidizable substrate, resulting in mineralization (De Coster et al. 2017).

Figure 2c, which presents the DOC *vs.* COD evolution lines for the experiments performed, well reflects the higher mineralization degree attained by BDD compared to Ti/MMO anodes. Higher slopes of these plots indicate higher mineralization rates (Fernandes et al. 2015). A slight increase in the mineralization rate with *j* is also noticed, which can be attributed to the enhanced formation rate of •OH with *j,* resulting in higher mineralization (Fernandes et al. 2015).

**Fig. 3:**
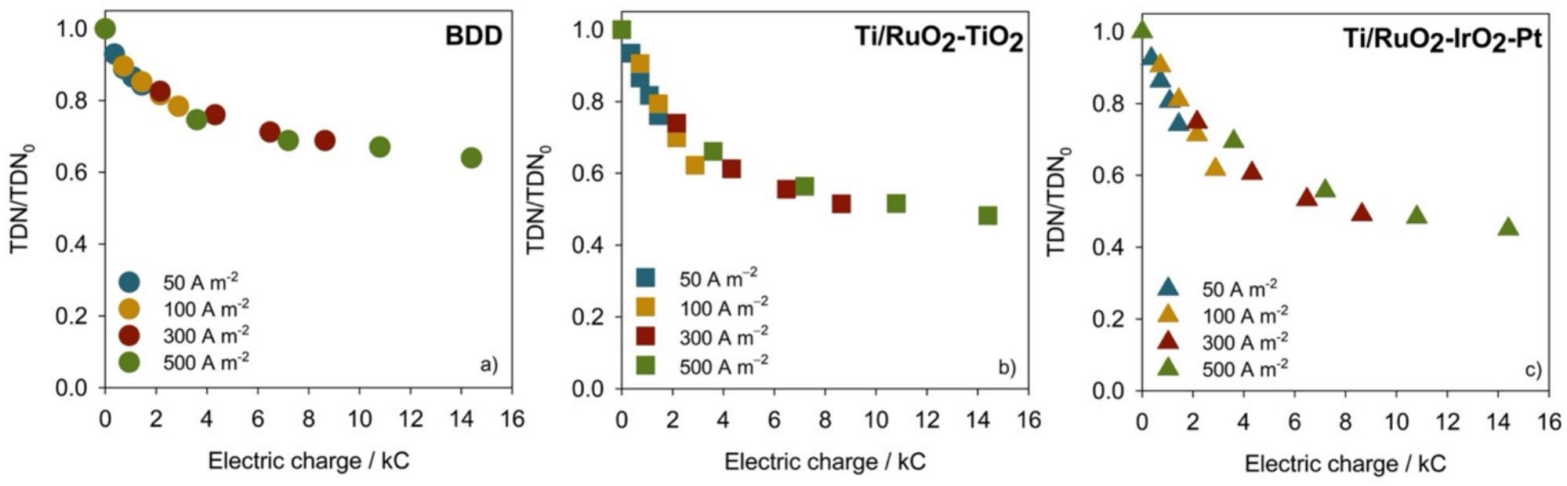
Normalized TDN decay with applied charge for the EO experiments performed at different applied current densities with different anode materials

Figure 3 presents the normalized TDN decay *vs.* electric charge for the EO experiments performed with different anode materials.

As observed for the COD and DOC decays, with the BDD anode, TDN removal follows a similar trend with the applied charge for the different current densities. On the other hand, for Ti/MMO anodes, a decrease in removal efficiency with the increase in *j* was observed, being more noticeable for Ti/RuO_2_-IrO_2_-Pt. This decrease in current efficiency with increased *j* is probably due to the faster oxidation of other compounds present in the solution at higher *j* (Baía et al. 2022). Nonetheless, for all the anodes studied, the TDN decay with time (data not shown) confirmed an increase in the removal rate with *j*, which is in accordance with the enhanced formation of oxidizing active species at higher currents that led to the faster oxidation of the nitrogen compounds for comparable treatment time.

The main results obtained from the different EO assays performed are shown in Table 2. After 8 h of treatment, the highest COD, TDC, and DOC removals were achieved using the BDD anode for all the studied *j*, indicating that BDD is more effective in oxidizing organic matter. Nevertheless, TDN removals were higher in the experiments performed with Ti/MMO anodes, as reported in different studies, and ascribed to the predominant oxidation of ammonium to nitrogen gas when metal oxide anodes are employed (Fernandes et al. 2014).

**Table 2:** Results obtained for the different parameters after 8 h of EO treatment, using BDD, Ti/RuO_2_-TiO_2_, and Ti/RuO_2_-IrO_2_-Pt anodes at different applied current densities

		BDD				Ti/RuO_2_-TiO_2_				Ti/RuO_2_-IrO_2_-Pt			
*j* (A m^−2^)		50	100	300	500	50	100	300	500	50	100	300	500
Removal (%)	COD	27	37	72	86	11	28	40	48	18	27	40	50
	TDC	11	18	43	65	1	2	3	7	1	2	5	11
	DOC	25	33	65	84	3	6	7	11	1	5	9	12
	TDN	16	18	31	36	20	38	47	54	24	38	49	55
DIC (mg L^−1^)		261	301	339	284	160	193	229	201	158	186	182	141
pH		8.01	7.82	7.72	9.85	10.7	9.93	8.71	9.25	10.4	9.79	9.22	10.8
E_EO_ (kWh m^−3^ order^−1^)		132	175	250	353	126	171	439	737	117	170	524	823

The literature suggests that ammonium degradation primarily occurs through indirect oxidation involving active chlorine species. These active chlorines can react with ammonium, prompting its oxidation (Pérez et al. 2012). The electrodes commonly used for in situ production of active chlorine are made of a mixture of metal oxides, known as MMO, that exhibit excellent electrocatalytic properties for chlorine evolution (Martínez-Huitle and Panizza 2018). This explains why Ti/MMO anodes are more efficient for nitrogen removal than BDD. Conversely, according to the literature, BDD is more effective at oxidizing ammonia to nitrate (Fernandes et al. 2014; Wilk et al. 2022).

The lowest *E*_EO_ value (117 kWh m^−3^ order^−1^) was also attained by a Ti/MMO anode, Ti/RuO_2_-IrO_2_-Pt, at the lowest applied *j*. Both Ti/MMO anodes presented promising results at *j* ≤ 100 A m^−2^, with electric energy per order consumptions slightly lower than BDD’s. This is explained by the lower voltage values the Ti/MMO anodes attained for equal applied *j*, as Ti/MMO anodes are more conductive than BDD (Fernandes et al. 2014). Still, for higher applied currents (*j* ≥ 300 A m^−2^), the lower potential difference attained by Ti/MMO anodes was not enough to compensate for the much lower COD removal compared to BDD, and, thus, the Ti/MMO *E*_EO_ values were higher than those obtained in the BDD experiments. The enhanced performance of BDD, especially at higher applied *j*, is ascribed to its high O_2_ overpotential. On the other hand, the decrease in the process efficiency with the increase in *j*, observed for the Ti/MMO anodes, has been explained by the enhancement of O_2_ production, which hinders the organic compounds oxidation (Silva et al. 2017). It is also worth noting that the EO operating conditions affect Ti/MMO anodes’ service life, with high current density being an important deactivation factor. According to the literature, RuO_2_-based anodes are susceptible to corrosion when oxygen evolves (Abdel-Aziz et al. 2024; Saha et al. 2020). This corrosion can lead to the deactivation of the anode, reducing its service life (Hoseinieh et al. 2010). Moreover, increased current may generate excess heat, resulting in thermal degradation of the electrode. The overheating can modify the physical and chemical properties of the Ti/MMO electrodes, consequently diminishing their effectiveness and service life. It is essential to optimize the current density to achieve a balance between degradation efficiency and electrode durability.

**Fig. 4:**
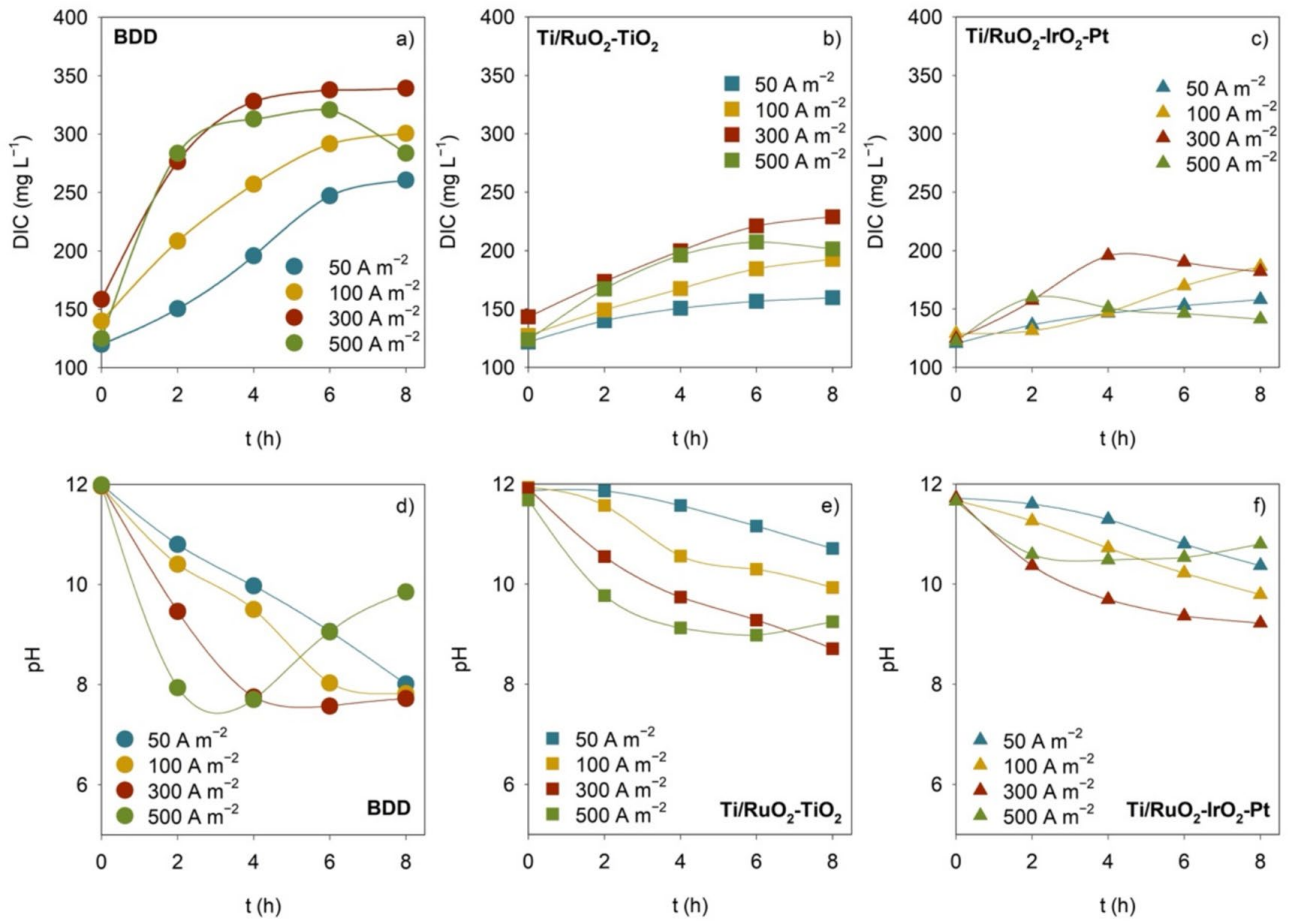
Variation with time of **a–c** DIC and **d–f** pH for the EO experiments performed with BDD, Ti/RuO_2_-TiO_2_, and Ti/RuO_2_-IrO_2_-Pt anodes at different applied current densities

For all the experimental conditions studied, an increase in DIC concentration and a decrease in pH were observed after 8-h treatment (Table 2). DIC and pH variations increased with *j* and were more pronounced in the experiments performed with BDD (Fig. 4). For all the anodes and *j* studied, the DIC formation rate was higher during the first hours of treatment, diminishing in the following hours. Indeed, at the highest applied *j*, a decrease in DIC concentration during the last hours of experiments was observed. The opposite trend was noticed for pH, with an initial decrease accentuated by the increase in *j* and an inversion in the initial decrease trend in the experiments run at the highest *j*. These findings suggest an inversely proportional relation between DIC and pH. Attending to the initial pH of the CWW sample (11.8) and that, in the pH range between 8.5 and 12, inorganic carbon is in the form of HCO_3_^−^ and CO_3_^2−^ (Ridgwell and Zeebe 2005), the DIC increase can be ascribed to the mineralization of the organic compounds (Eqs. (6) to (8), where *M* denotes the anode, and *R* represents the organic compounds). This follows the higher mineralization degree and DIC formation observed for BDD, compared to Ti/MMO anodes.

6 $$\text{M}\left(\cdot \text{OH}\right)+\text{R}\to \text{M}+{\text{CO}}_{2}+{\text{H}}_{2}\text{O}+{\text{H}}^{+}+{\text{e}}^{-}$$

7 $${\text{CO}}_{2}+{\text{HO}}^{-}\leftrightarrow {{\text{HCO}}_{3}}^{-}$$

8 $${{\text{HCO}}_{3}}^{-}\leftrightarrow {{\text{CO}}_{3}}^{2-}+{\text{H}}^{+}$$

The release of H^+^ from reactions (6) and (8) and from other redox reactions that may occur, and the carboxylic acid formation from the organic matter oxidation, can explain the observed decrease in pH when DIC concentration is increasing (Souli et al. 2023). These reactions are enhanced with the increase in *j* and with BDD utilization. The decrease in pH should, in turn, force the chemical re-equilibrium of the DIC system, which can result in the release of CO_2_ and, consequently, in the decrease of DIC concentration. Moreover, the reaction between HCO_3_^−^ and active chlorine species (Eq. (9)) can also explain the reduction in DIC concentration (Fernandes et al. 2020).

9 $$\text{HOCI}+{{\text{HCO}}_{3}}^{-}\leftrightarrow {\text{CO}}_{2}+{\text{OCI}}^{-}+{\text{H}}_{2}\text{O}$$

The suppression of some of the reactions that lead to H^+^ in solution and the enhancement of secondary reactions, as the reaction of hydrogen evolution, caused, for instance, by the reduction in oxidizable compounds, can explain the pH increase when DIC is decreasing (Souli et al. 2023).

**Fig. 5:**
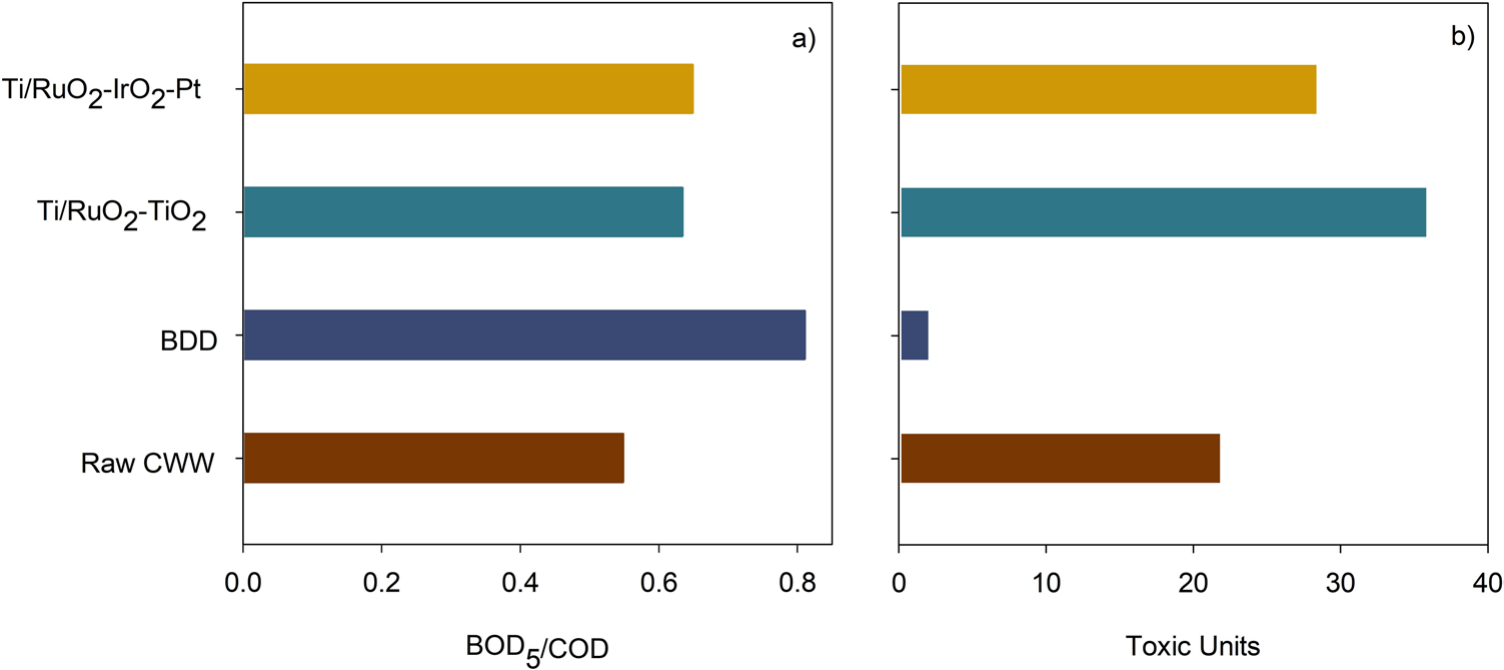
**a** Biodegradability and **b** ecotoxicity results, after 8 h of EO treatment, using BDD, Ti/RuO_2_-TiO_2_, and Ti/RuO_2_-IrO_2_-Pt anodes, at an applied *j* of 500 A m^−2^

Due to the diversity of compounds in real industrial wastewater, the toxicity and biodegradability index of treated wastewater are important considerations for the successful application of EO treatment. Figure 5 presents the biodegradability and ecotoxicity towards *Daphnia magna* of the CWW samples treated for 8 h at 500 A m^−2^, using the different anode materials under study.

BDD anode presented the best results, with an increase in biodegradability, given as the BOD_5_/COD ratio, from 0.55 to 0.81, and a reduction in the ecotoxicity towards *Daphnia magna* from 21.7 to 1.87 TU, which changed the CWW classification from “very toxic” (10 < TU < 100) to “toxic” (1 < TU < 10), very close to the “non-toxic” level (TU < 1) (Pablos et al. 2011). According to the literature, the remaining ecotoxicity of the treated CWW can be due to oxidation end-products, such as acetic and propionic acids, that are toxic to *Daphnia magna* (Baía et al. 2022). These results are under the high oxidation degree of the degradation products achieved with this anode.

For Ti/MMO anodes, the biodegradability increased from 0.55 to approximately 0.64, indicating that the products of the EO process are more biodegradable than the original CWW constituents. On the other hand, the ecotoxicity results show, for Ti/MMO anodes, an increase in the toxic units of the treated effluents, revealing that they are more toxic to the model organism than the untreated one. This increase in ecotoxicity during EO treatment using Ti/MMO anodes is reported in the literature (Wu et al. 2014; Radjenovic et al. 2011). It is attributed to the production of degradation products that are more toxic than the original pollutants, such as organochlorinated compounds and chloramines (Fernandes et al. 2019; Wu et al. 2014). According to the literature, organochlorinated compounds can be formed during EO whenever electrolysis is performed in chloride media, increasing the toxicity of the wastewater (Panizza and Cerisola 2003). Markou et al. (2017) reported that the EO treatment of dairy wastewater containing high concentrations of sodium chloride, using dimensionally stable anodes, can lead to treated wastewater with high toxicity due to the formation of organochlorides. These authors noticed that, in the presence of sodium chloride, the evolution of compounds absorbing at the wavelength of 280 nm (associated with organochlorinated molecules) was much higher than that when sodium sulfate was used. In a different study, Katsoni et al. (2014) identified, during the EO treatment of cheese whey, several organochlorinated compounds, namely, trihalomethanes, haloacetonitriles, and haloketons, as well as 1,2-dichloroethane and chloropicrin. According to the authors, these compounds are responsible for the increased ecotoxicity observed for the EO treatment. The CWW sample utilized in the present study had a chloride concentration of approximately 800 mg L^−1^, which, in addition to the incomplete oxidation of the organic compounds promoted by the Ti/MMO anodes, fits the ecotoxicity increase observed for these anodes. Although the TDN concentration (≈ 91 mg L^−1^) was much lower than that of chloride, the formation of chloramines, considered very toxic degradation products, cannot be disregarded. According to the literature, chloramines can be formed by the presence of ammonia and hypochlorous acid in solution, according to Eqs. (10) to (12) (Pérez et al. 2012; Souli et al. 2023).

10 $${\text{NH}}_{4}^{+}+\text{HOCl }\to {\text{NH}}_{2}\text{Cl}+{\text{H}}_{2}\text{O}+ {\text{H}}^{+}$$

11 $${\text{NH}}_{2}\text{Cl}+\text{HOCl }\to {\text{NHCl}}_{2}+{\text{H}}_{2}\text{O}$$

12 $${\text{NHCl}}_{2}+\text{HOCl }\to {\text{NCl}}_{3}+{\text{H}}_{2}\text{O}$$

Ti/RuO_2_-TiO_2_ anode presented a more pronounced increase in ecotoxicity than Ti/RuO_2_-IrO_2_-Pt. Although these two anodes presented similar performance in terms of the main parameters analyzed, the COD, TDC, DOC, and TDN removals were, in fact, slightly lower for the Ti/RuO_2_-TiO_2_ anode. This slight difference in the performance of the two Ti/MMO anodes can be attributed to the IrO_2_ and Pt layers at Ti/RuO_2_-IrO_2_-Pt, as described above.

The electrochemical oxidation, using BDD and Ti/MMO anodes, was very effective in decolorizing the cheese whey wastewater, particularly at the highest applied *j* (500 A m^−2^). EO efficacy in reducing CWW coloration has been established in prior literature (Katsoni et al. 2014). It is attributed to the high bleaching properties of active chlorine, formed from chloride oxidation, which also promotes water disinfection, eliminating pathogenic microorganisms, and unpleasant smells (Garcia-Segura et al. 2015; Panizza et al. 2007).

Since EO utilizing BDD anode successfully minimizes the ecotoxicity of treated CWW (nearly reaching the “non-toxic” level) and enhances the biodegradability index, the coupling of the EO-BDD process with a biological treatment can be a possible solution to eliminate residual pollution and to make the effluent comply with discharge limits (Ganzenko et al. 2014). Experimental conditions should be tailored to the desired objectives. If the priority is N-containing compound removal over organic load removal or if the sample has a high nitrogen content, EO-Ti/MMO processes should be employed due to their lower cost than BDD. Experimental conditions must be optimized to achieve complete degradation/mineralization yields while avoiding the formation of unwanted toxic compounds.

## Conclusion

The efficiency of electrochemical oxidation in purifying CWW was investigated utilizing various anode materials and applied current densities. Higher current densities resulted in heightened rates of pollution removal. The BDD anode exhibited superior COD, TDC, and DOC removal performance at various current densities, whereas the Ti/MMO anodes had higher TDN removal efficiency. At a current density of 500 A m^−2^, the use of BDD anode resulted in an improvement in biodegradability (as indicated by an increase in the BOD_5_/COD ratio from 0.55 to 0.81) and a reduction in ecotoxicity towards *Daphnia magna* (from 21.7 to 1.87 TU). Utilizing Ti/MMO anodes increased the biodegradability index to ≈ 0.64, but the ecotoxicity also increased. The increase in ecotoxicity with Ti/RuO_2_-TiO_2_ anode was more pronounced than with Ti/RuO_2_-IrO_2_-Pt, despite both anode types demonstrating identical performance in the primary analysis parameters. Both Ti/MMO anodes demonstrated encouraging outcomes when operated at current densities equal to or below 100 A m^−2^. Ti/RuO_2_-IrO_2_-Pt presented the lowest electric energy per order consumption (117 kWh m^−3^ order^−1^) when operated at the lowest current density (50 A m^−2^). Nevertheless, when current densities reached 300 A m^−2^ or more, the *E*_EO_ values of Ti/MMO anodes surpassed those observed in BDD studies.

## Data Availability

All data obtained have been included into the manuscript and available from the corresponding author upon reasonable request.
